# Pragmatics prevail over paradigms in a war of supremacy for physical education

**DOI:** 10.3389/fspor.2025.1643738

**Published:** 2025-07-01

**Authors:** Dean Alan Dudley

**Affiliations:** ^1^ School of Human Movement and Nutrition Sciences, University of Queensland, Brisbane, QLD, Australia; ^2^ Centre for Educational Measurement and Assessment, University of Sydney, Sydney, NSW, Australia; ^3^ Performance and Expertise Research Centre, Macquarie University, Sydney, NSW, Australia

**Keywords:** renaissance, conflict, interdiscipinarity, cooperation, change management

## Introduction

I have always been intrigued by the Renaissance era of human civilisation. If you are ever unfortunate enough to step into my lectures, you will see students suffering from my endless presentation slides littered with the works of Leonardo Da Vinci and a rhetoric rejecting the Cartesian dualism of the body and the mind. For me and many others, the Renaissance era represented a pivotal moment in human civilisation, celebrated for its flourishing of intellectual, artistic, and scientific innovation. Notably, this period of human evolution marked a departure from the rigid scholasticism of the medieval epoch, which had been largely dominated by theological dogma and Aristotelian paradigms mediated through ecclesiastical authority. Instead, the Renaissance ushered in a spirit of inquiry characterised by empirical curiosity, humanistic values, and a pragmatic orientation towards knowledge production and application. This shift laid the groundwork for a mode of innovation that privileged utility, observation, and adaptability over allegiance to inherited truths or fixed epistemologies.

Central to this transformation was the rise of humanism, a philosophical and cultural movement that emphasised the potential of human beings to shape and understand the world through reason, experience, and creativity (1). Renaissance humanists such as Petrarch, Erasmus, and later figures like Francis Bacon, championed the study of classical texts not as dogma to be preserved, but as sources of critical insight and tools for living. This recontextualisation of ancient knowledge enabled scholars and practitioners to adopt eclectic research methods, drawing from diverse intellectual traditions in ways that were responsive to contemporary problems rather than constrained by theological orthodoxy or scholastic systems.

Figures such as Leonardo da Vinci, Galileo Galilei, and Andreas Vesalius exemplified an empirical turn in knowledge production. The use of intrinsic and humanistic learning traits such as observing, dissecting, experimenting, and drawing conclusions grounded in material experience rather than metaphysical speculation were celebrated. Da Vinci's notebooks, for example, seamlessly wove together artistic, anatomical, and engineering insights, resisting categorisation within a single discipline or worldview. Such polymathic endeavours were enabled by a sociocultural milieu that valued *ad hoc* synthesis over paradigmatic purity. In this way, the Renaissance can be seen not as a triumph of one intellectual framework over another, but as a fertile period of epistemic openness in which innovation emerged from the generative interplay of ideas, practices, and disciplines.

This pragmatism also had practical implications in understandings of human movement and the human experience. Renaissance-informed epistemology reveals compelling synergies that illuminate how physicality, embodiment, and kinaesthetic knowledge were central, not peripheral, to the innovation and intellectual flourishing of the era. Far from being abstract or disembodied, the Renaissance's pragmatic orientation to knowledge was deeply rooted in lived experience, material engagement, and the sensuous dimensions of the human condition. This embodied turn resonates with contemporary perspectives in physical education and pedagogy, particularly those informed by phenomenology and ecological dynamics.

The work of Renaissance polymaths, like da Vinci, exemplified how the study of human movement was both empirical and expressive. Da Vinci's anatomical investigations that were meticulously documented through dissection and observational drawing were not simply scientific, they represented a profound inquiry into the mechanics and aesthetics of the moving body. His Vitruvian Man, synthesising art, science, and proportion, symbolises a human-centric cosmology in which bodily knowledge was a source of truth in and of itself. Here, the human body was not merely a passive subject of study but an active site of discovery, a notion that anticipates contemporary understandings of movement as a form of embodied cognition (2).

The Renaissance's revival of classical ideals also placed renewed emphasis on *paideia*, the cultivation of the whole person, including physical, moral, and intellectual capacities. The gymnasium, resurrected from Greco-Roman traditions, became a space for the holistic development of youth, where movement practices were valued not only for their health benefits but also for their formative role in shaping character, discipline, and social responsibility. Such views reinforce the idea that the human experience of movement is not reducible only to performance metrics or physiological outputs, but is also fundamentally relational, situational, and existential.

Furthermore, Renaissance dance, theatre, and martial arts were pedagogical in nature, often serving as vehicles for exploring rhythm, control, emotional expression, and cultural identity. These practices demonstrate an understanding of movement as a communicative and affective medium. This is a perspective echoed in modern theories of physical literacy, which foreground the body as a site of learning, adaptation, and meaning-making (3).

### Renaissance in ruins

Fast forward to the contemporary field of Physical Education which has, for decades at least, been characterised by intense theoretical contestation and competing paradigms. They have shaped, and often fractured, its identity, aims, and pedagogical orientations. These so-called “paradigm wars” (epitomized by the infamous Schempp vs. Siedentop dual and McKenzie vs. Gard ‘I don’t read fiction’ confrontation of the 1980's and 2000's) have played out across philosophical, political, and methodological lines, with factions variously advocating for performance-based models, critical pedagogies, health-focused curricula, and more recently, sociocultural and ecological perspectives on movement and embodiment. While such contestation has arguably stimulated intellectual growth and diversified pedagogical approaches, it has also contributed to fragmentation within the discipline, often impeding the development of coherent research agendas and unified advocacy for the field within broader educational and policy frameworks.

Contemporary and historical paradigms alike often confine scholarly inquiry within rigid epistemological and ontological binaries causing reality to be framed as either entirely objective or wholly socially constructed. Such dichotomies obscure more nuanced, mediated positions that acknowledge reality as existing independently of human perception yet invariably accessed and interpreted through socially constructed filters. This perspective recognises that while reality has an objective basis, our understanding of it is shaped by cultural, historical, and linguistic contexts. Scholars can explore this duality through thought experiments or empirical investigations, such as comparative analyses across different time periods or societies, to reveal how both objective and socially constructed dimensions of reality coexist. Nevertheless, many academics appear compelled—whether by disciplinary conventions, institutional pressures, or the seductive simplicity of binaries—to align themselves with one pole or the other. This raises critical questions about the intellectual and structural forces that discourage more integrative epistemological positions within academic discourse.

At the core of ongoing paradigm conflicts in physical education lies a fundamental and enduring question: *What is the purpose of physical education?* Is it primarily a vehicle for enhancing physical performance and fitness, a means of promoting lifelong engagement in physical activity, or a platform for social transformation and identity development through movement? Pate and Hohn (4) famously referred to this uncertainty as a “muddled mission,” capturing the lack of consensus within the field. Indeed, physical education has been so frequently reshaped to meet shifting societal expectations that McKenzie & Lounsbery (5) labelled it the “chameleon of all curricula.” This continual redefinition reflects deeper philosophical tensions that, in many ways, echo the existential nature of asking, “What is the meaning of life?”. The current climate surrounding this question has fostered a combative dynamic reminiscent of *Game of Thrones*. We exist in a discipline with paradigmatic factions forming strategic alliances and engaging in intellectual battles to assert the primacy of their worldview of physical education and pedagogy. Rather than clarifying the field's direction, such conflict often entrenches division and impedes collective progress.

Despite calls for a truce to this conflict (6), the field continues to grapple with divergent conceptualisations of knowledge, practice, and purpose. These unresolved tensions are further exacerbated by the demands of rapidly evolving educational landscapes, the global rise of non-communicable disease, growing attention to youth mental health and wellbeing, and the challenges posed by digital and post-pandemic schooling environments. Collectively, these forces necessitate a reimagining of what Physical Education research can, and should, prioritise.

### Renaissance reignited

This article sets out an ambitious agenda for the *Physical Education and Pedagogy* speciality of *Frontiers in Sports and Active Living*, grounded in a commitment to both scholarly pluralism and meaningful impact. Rather than attempting to resolve longstanding ideological disputes, this agenda invites generative dialogue and transdisciplinary engagement across paradigmatic boundaries to provide the fertile garden of epistemic openness for a renaissance of thought to flourish. Justification for fostering generative dialogue and transdisciplinary engagement in physical education rather than attempting to resolve paradigm disputes can be found in several key developments in the field's recent literature, research methodologies, and evolving practice environments.

Numerous bibliometric analyses and systematic reviews [e.g. (7–10)] reveal a marked increase in scholarship that defies conventional paradigmatic categorisation. These studies show that many emerging physical education researchers adopt hybrid theoretical frameworks, drawing simultaneously from critical pedagogy, post-structural theory, ecological dynamics, and biophysical approaches. This suggests an emergent epistemological pragmatism, where scholars prioritise the utility of concepts and methods over allegiance to singular paradigms. Rather than being evidence of theoretical incoherence, this methodological pluralism reflects a field attuned to the complex and context-specific nature of educational practice. Attempts to impose epistemological closure may therefore be counterproductive, stifling innovation and marginalising valuable insights from non-dominant perspectives (11).

Furthermore, research suggests that pedagogical approaches grounded in transdisciplinary thinking can effectively address the multifaceted realities of students’ lives (12). For instance, pedagogies that integrate physical activity with identity exploration, social justice, and digital literacies have shown promise in engaging students who are traditionally marginalised or disengaged from physical education (13, 14). These findings underscore the value of research that transcends disciplinary silos and foregrounds the lived experiences of learners. They also highlight how generative dialogue across paradigms can yield pedagogical innovations that are more inclusive, responsive, and contextually grounded than those derived from monolithic theoretical commitments.

We also find evidence from collaborative action research projects and practitioner-led inquiry (15, 16) that demonstrates the transformative potential of participatory methodologies that bring together researchers, teachers, students, and community stakeholders. These initiatives often necessitate crossing paradigmatic and disciplinary boundaries, as they involve negotiating multiple forms of knowledge (i.e., experiential, theoretical, cultural, and embodied). The success of such projects in effecting sustainable pedagogical change provides a compelling empirical rationale for fostering research cultures that privilege dialogue over dogma.

Collectively, these strands of evidence pave a future of Physical Education scholarship that lies not in the resolution of its past ideological conflicts, but in cultivating the conditions (much in the spirit of the Renaissance) for collaborative, pluralistic, and impact-oriented inquiry. By embracing generative dialogue and transdisciplinary engagement, the field can move beyond entrenched divides to better serve diverse learners and communities in an increasingly complex world.

In the pursuit of research that is responsive to the complex realities of contemporary schooling, culturally attuned, methodologically diverse, and globally conscious. We aim to reposition the *Physical Education and Pedagogy* section not as a battleground for theoretical supremacy, but as a collaborative platform for advancing knowledge that is both academically rigorous and socially relevant.

Through this agenda, we invite contributions that address enduring and emergent challenges in Physical Education, encourage innovation in pedagogical design and delivery, and critically examine the sociopolitical contexts in which movement and learning occur. By embracing complexity and fostering intellectual generosity, we hope to cultivate a more connected, coherent, and impactful future for Physical Education scholarship.
